# Surgical resection of solid gallbladder adenocarcinoma presenting as a large mass: report of a case

**DOI:** 10.1186/s12957-014-0416-2

**Published:** 2015-02-07

**Authors:** Satoshi Hayama, Satoshi Hirano, Nagato Sato, Yuma Ebihara, Yo Kurashima, Soichi Murakami, Eiji Tamoto, Toru Nakamura, Joe Matsumoto, Takahiro Tsuchikawa, Eiichi Tanaka, Toshiaki Shichinohe

**Affiliations:** Department of Gastroenterological Surgery II, Hokkaido University Graduate School of Medicine, N-15, W-7, Kita-ku, Sapporo, Hokkaido 085-8512 Japan

**Keywords:** Clinicopathological feature, Gallbladder, Solid adenocarcinoma, Surgical treatment, Tumor thrombus

## Abstract

This report describes a case of a patient with a large solid gallbladder adenocarcinoma that was completely resected through aggressive surgery. The patient was a 57-year-old woman who had been diagnosed with advanced gallbladder cancer, had no indications for surgical resection and was scheduled to undergo systemic chemotherapy. She presented to our hospital for a second opinion. At the time of assessment, her tumor was large but was well-localized and had not invaded into the surrounding tissues, indicating that surgical resection was a reasonable option. Subsequently, the tumor was completely extracted via right hepatectomy with *en bloc* resection of the caudate lobe and extrahepatic bile duct. Histopathologically, the tumor was a solid adenocarcinoma. Although there are relatively few reports in the literature regarding solid gallbladder adenocarcinoma, well-localized growth appears to be a characteristic feature. On the basis of a tumor’s progression behavior, aggressive surgical treatment might be indicated even when the tumor has grown to a considerable size.

## Background

Some gallbladder cancers (GBCs) do not show invasive behavior, even when the tumors grow large, and these types of tumors are associated with better outcomes than tumors that do show invasive behavior. Surgery is the only curative modality for GBC, whereas nonsurgical therapies (for example, chemotherapy (cisplatin and gemcitabine) as first-line chemoradiotherapy) can provide limited survival benefit [1]. Therefore, it is of critical importance to detect these well-localized GBCs via imaging and to proceed with appropriate surgical resection. Elevated expression of CDX2 and hepatocyte antigen (Hep) in GBC is associated with less aggressive behavior [2], and activating mutations in *KRAS* are associated with more malignant behavior [1,3,4]. Use of this molecular information might allow for better estimation of prognosis and to help guide personalized surgical therapy.

## Case presentation

**Figure 1:**
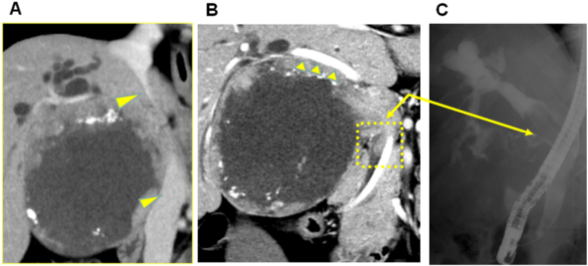
**Abdominal computed tomography scans reveal a large, centrally necrotizing and well-demarcated tumor. (A)** The tumor compressed the neighboring tissue Yellow arrowheads indicates the inferior vena cava. **(B)** Yellow arrowheads indicate the right intrahepatic Glisson. **(C)** Endoscopic retrograde cholangiopancreatography reveals that the tumor extended to the intrapancreatic bile duct (arrows in **B** and **C**).

A 57-year-old woman presented to a hospital other than ours, complaining of icterus. She was diagnosed with advanced GBC. The surgeons there thought an operation was not indicated, and she was scheduled to be initiated on systemic chemotherapy. However, she presented to our hospital for a second opinion. Abdominal computed tomography demonstrated a large tumor in the neck and body of the gallbladder, with expansive growth and compression of the portal vein, common bile duct (CBD), liver bed and inferior vena cava. However, the tumor did not invade into these tissues (Figure 1A,B). The tumor extended to the bile duct, resulting in obstructive jaundice; therefore, the likely cause of tumor extension was tumor thrombus, cancer invasion or longitudinal tumor spread along the biliary tree. Regardless, preoperative differentiation between these possibilities was impossible (Figure 1C). There was no sign of metastasis to the liver, peritoneum, or paraaortic lymph nodes. Taken together, these radiological findings suggested that this large tumor was potentially resectable.

A right hepatic lobectomy with extrahepatic bile duct resection was considered to be the most appropriate surgical procedure. Therefore, to prevent postoperative liver failure, preoperative right portal vein embolization (PVE) was performed, and the patient underwent radical surgery 3 weeks after PVE.

**Figure 2:**
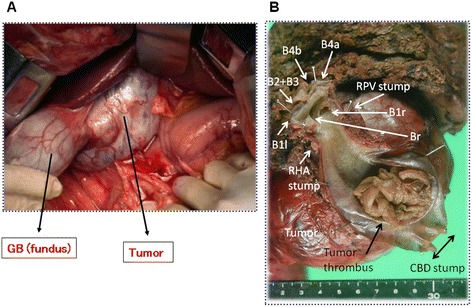
**Intraoperative findings.** The large and soft gallbladder (GB) tumor was palpable **(A)**. The tumor was completely removed via right hepatic lobectomy with *en bloc* resection of the caudate lobe and extrahepatic bile duct **(B)**. B1(r and l), Caudate lobe duct (right and left); B2 and B3, Lateral superior and inferior ducts; B4 (a and b), Medial segmental duct (inferior and superior branches); Br, Right hepatic duct; CBD, Common bile duct; RHA, right hepatic artery; RPV, Right portal vein.

At laparotomy, a large, soft tumor was palpable in the neck and body of the gallbladder. The tumor compressed, but did not seem to infiltrate, the surrounding gallbladder tissues (Figure 2A). A right hepatic lobectomy with *en bloc* resection of the caudate lobe and the extrahepatic bile duct was performed. Because of tumor extension, the extrahepatic duct was resected up to the intrapancreatic portion, and, on the hepatic side, hepatic duct was resected to the most peripheral point where the hepatic ducts could be separated from the vasculature during the right hepatectomy. This is the limit of ductal resection [5] to ensure negative margins (Figure 2B).

**Figure 3:**
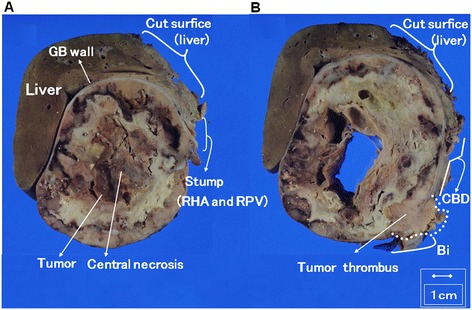
**Macroscopic view of the cut specimen revealed a centrally necrotizing tumor that was 10.7 × 10 cm in size.** The patient’s gallbladder (GB) tumor compressed adjacent tissues and organs but did not invade into them **(A)**. RHA, right hepatic artery; RPV, Right portal vein. Tumor necrotic tissue that extended to the intrapancreatic bile duct (Bi) was also completely resected **(B)** CBD, Common bile duct.

The macroscopic view of the cut specimen revealed a large tumor with central necrosis and measuring 10.7 × 10 cm in size. The resected tumor was well-demarcated and did not invade into the surrounding tissues. The tumor necrotic tissue extended from the tumor up to the intrapancreatic bile duct, which was also completely resected (Figure 3A,B).

**Figure 4:**
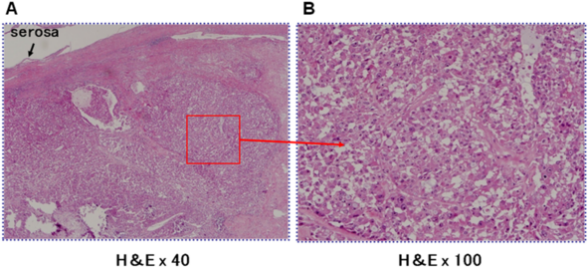
**Photomicrographs showing the histopathologic appearance of atypical cells with an eosinophilic granular cytoplasm.** These cells formed solid nests (hematoxylin and eosin stain (H&E); original magnification, ×40) **(A)**. The cells had a large and eccentric nucleus and a prominent nucleolus (H&E stain; original magnification, ×100) **(B)**.

Histopathologically, atypical cells with an eosinophilic and granular cytoplasm formed solid nests without tubular structures. These cells had a large and eccentric nucleus and a prominent nucleolus (Figure 4A,B). Immunohistochemically, the tumor cells were positive for Hep and pan-cytokeratin, but negative for α-fetoprotein. The highest Ki-67 index was 20% in the tumor. Tumor invasion was limited to the subserosal layer, and no vascular invasion or perineural invasion was identified. The necrotic tissue extended to the biliary tract via the cystic duct, but no tumor cells replaced epithelial cells of the cystic duct or biliary tract. Therefore, a diagnosis of tumor thrombus extending from the tumor was made. The histopathologic diagnosis was solid adenocarcinoma of the gallbladder, and *en bloc* R0 resection was confirmed histopathologically. The tumor-node-metastasis classification according to the Union for International Cancer Control [6] system was pT2, pN0, M0, pStage IB.

The patient had an uneventful postoperative recovery and was discharged from the hospital on the 22nd postoperative day. She received neither adjuvant chemotherapy nor radiation therapy. Two years after the operation, she was in good health, with no signs of recurrence.

## Discussion

Solid adenocarcinoma of the gallbladder, originally classified as a category of poorly differentiated adenocarcinoma, has been reclassified as a distinct entity according to the Japanese Society of Biliary Surgery classification system (4th edition) [7]; however, this neoplasm has not been classified by the World Health Organization [8]. Histopathologically, this neoplasm is composed of tumor cells with atypical nuclei and an eosinophilic cytoplasm, and these cells form solid nests [9].

In the present case, the patient had a large tumor with marked central necrosis and expansive growth. In general, these features are typical of undifferentiated gallbladder carcinoma [10]. Surgical treatment is not likely to be curative for such a large, undifferentiated carcinoma, as these tumors are often associated with distant metastasis or a locally advanced tumor. In fact, the 1-year survival rate for advanced, undifferentiated gallbladder carcinoma is only 18% [11]. Therefore, diagnostic differentiation of undifferentiated gallbladder carcinoma and other histological types is essential.

The tumor in our patient had radiological features that made it difficult to distinguish from undifferentiated carcinoma; consequently, surgery was not initially thought to be indicated. However, detailed review of the radiological and intraoperative findings showed that the tumor had not invaded to nearby tissues, indicating that radical surgery was an appropriate management option. Such expansive tumor growth with a well-demarcated margin is a characteristic feature of solid gallbladder adenocarcinoma, as described in several reports and presentations [12,13].

The histological type of GBC is a significant predictor of outcome. For example, papillary adenocarcinoma and well-differentiated adenocarcinoma are associated with better outcomes than undifferentiated adenocarcinoma and poorly differentiated carcinoma [11,14,15]. Further, solid gallbladder adenocarcinoma is associated with better postoperative outcomes than poorly differentiated gallbladder adenocarcinoma. Although there has been only one study with regard to survival for solid gallbladder adenocarcinoma, the reported 1- and 3-year survival rates were 53.8% and 17.9%, respectively, for solid gallbladder adenocarcinoma and 37.5% and 0%, respectively, for poorly differentiated gallbladder adenocarcinoma [16]. Moreover, a solid adenocarcinoma may show a spectrum of malignancy because it is sometimes heterogeneous and contains foci of poorly differentiated adenocarcinoma [9]. Therefore, in pure solid gallbladder adenocarcinoma (as in our patient), favorable postoperative outcomes would be expected. However, for more proper indicators of the surgical prognosis in this neoplasm, a better comprehension of cellular and molecular pathogenesis is required, which could also lead to the potential utility of targeted therapies, as reported in *PIK3CA* and *ALK* mutations [3,4]. To date, elevated expression of CDX2 and Hep have been reported to be associated with GBC of less aggressive behavior [2], whereas *KRAS* mutation has been reported to be associated with poor survival [4]. Interestingly, Hep was positive in our patient. Accrual of additional analyses of Hep expression will reveal the relatively low malignant nature of this neoplasm.

Finally, GBCs with tumor thrombus in the CBD are very rare [17,18]. Including the present case, two of three cases of solid gallbladder adenocarcinoma described in the literature were accompanied by tumor thrombus in the CBD, which suggests that this finding may be a characteristic feature of this neoplasm. However, preoperative differential diagnosis of tumor thrombus due to cancer invasion or intraepithelial cancer spread along the biliary tree remains difficult. In patients with intraepithelial cancer spread, the margin of the cancer is sometimes hard to characterize preoperatively. Therefore, to ensure ductal negative margins in our patient, the extrahepatic duct was resected as peripherally as possible.

## Conclusions

In this report, we describe a case of a patient with large, centrally necrotizing but well-localized GBC. Clinicians should be aware that some GBCs do not show invasive behavior, even when the tumors grow large in size, and that these tumor types are associated with better outcomes than tumors that do show invasive behavior. Solid gallbladder adenocarcinoma likely constitutes one of the histologic types of such a resectable GBC. As in the present case, complete resection of a pure solid gallbladder adenocarcinoma can presumably lead to long-term survival. More detailed analysis of the molecular pathogenesis of gallbladder adenocarcinoma might enable characterization of the clinicopathological features of this neoplasm and the potential utility of targeted therapies.

## Consent

Written informed consent was obtained from the patient for publication of this case report and any accompanying images. A copy of the written consent is available for review by the Editor-in-Chief of this journal.

## Abbreviations

Bi: Intrapancreatic bile duct
Br: Right hepatic duct
CBD: Common bile duct
GB: Gallbladder
GBC: Gallbladder cancer
Hep: Hepatocyte antigen
PVE: Portal vein embolization
RHA: Right hepatic artery
RPV: Right portal vein
